# For What Illnesses Do Asylum Seekers and Undocumented Migrant Workers in Israel Seek Healthcare? An Analysis of Medical Visits at a Large Urgent Care Clinic for the Uninsured in Tel Aviv

**DOI:** 10.3390/ijerph16020252

**Published:** 2019-01-16

**Authors:** Elizabeth B. Moran, Mark A. Katz, Orel-Ben Ari, Nadav Davidovitch, Oren Zwang

**Affiliations:** 1 University of Michigan, School of Public Health, Ann Arbor, MI 48104, USA; ebmoran@umich.edu (E.B.M.); katzmar@post.bgu.ac.il (M.A.K.); 2 Terem Urgen Care, Jerusalem 91000, Israel; orelbenari@gmail.com; 3 Ben Gurion University of the Negev, School of Public Health, Beer-Sheva 84102, Israel; nadavd@bgu.ac.il

**Keywords:** refugee, health, migration, chronic disease, infectious disease

## Abstract

In 2017, there were nearly 80,000 asylum seekers and undocumented migrant workers in Israel, most of whom did not have health insurance. We evaluated trends in medical visits of asylum seekers and undocumented migrant workers who presented to Terem Refugee Clinic (TRC), a large clinic in Tel Aviv available only to uninsured residents of Israel. Data were collected from electronic medical records at TRC from 2013–2017. Diagnoses were grouped into categories using ICD-10-equivalent diagnosis codes. We used a chi-squared test for trends to test the significance of trends 2013 to 2017. There were 99,569 medical visits from 2013 to 2017 at TRC. Visits were lowest in 2013 (11,112), and relatively stable from 2014–2017 (range: 19,712–23,172). Most visits were among adults aged 18–35 (41.2%) and children <2 years old (23.7%). Only 3% of visits were from patients aged >50. The percentage of infectious disease diagnoses decreased over the study period, from 9.4% of all diagnoses in adults in 2014 to 5.2% in 2017, and from 32.0% of all diagnoses in children in 2013 to 19.4% in 2017. The annual percentage of respiratory diagnoses in children and adults 18–35 years of age, musculoskeletal in all adults, and digestive in adults except women ≥35 years old increased. Over time, asylum seekers and undocumented migrant workers visited TRC with fewer infectious diseases diagnoses overall but more respiratory diseases, including acute respiratory infections and more musculoskeletal diseases.

## 1. Introduction

The number of international migrants worldwide has risen significantly in recent years and now totals approximately 258 million people, which includes 25.4 million refugees and 3.1 million asylum seekers [1]. Both refugees and asylum seekers flee their country because of persecution, war or violence. Refugees have been granted legal status and receive protection and assistance from their host country, whereas asylum seekers are awaiting legal recognition as refugees [2]. Migrant workers are persons who are employed in a country of which they are not a citizen. Undocumented migrant workers do not have legal permission to reside and work in their host country and do not benefit from legal protections [3]. Asylum seekers tend to include families and individuals of a range of ages and undocumented migrant workers tend to be young men [2,3]. The health of these refugees, asylum seekers, and undocumented migrant workers is crucial for reasons including human rights, public health, the economic stability of their communities, and the stability of the healthcare systems in the countries in which they reside [4]. Refugees and other immigrants tend to arrive to new countries mostly healthy, but their health often deteriorates over time—a pattern referred to as the “healthy immigrant effect” that has been described in several countries, including in North America and in Europe [5,6,7,8]. In addition, over time, the health problems of refugees and asylum seekers tend to shift from mostly acute infectious illnesses to chronic diseases, such as cardiovascular disease and diabetes [5].

In the last decade, Israel has experienced a marked increase in the number of asylum seekers and undocumented migrant workers. From 2006 to 2012, over 60,000 asylum seekers, primarily from Eritrea and South Sudan, arrived in Israel by way of the Sinai Peninsula, and as of the end of 2017, 35,659 were still living in the country, most of whom were residing in South Tel Aviv [9,10,11,12]. In addition, according to the Israeli Population and Immigration Authority, by the end of June 2018, an estimated 19,250 migrant workers were living in Israel after their initial visas and work-related health insurance had expired [10]. Asylum seekers are not covered under Israel’s national health insurance fund, but they have the option to buy private health insurance. Many asylum seekers do not enroll in these plans due to the high cost. Legal migrant workers are required to have health insurance, whereas undocumented migrant workers often do not have health insurance.

While refugee health has been studied in high-income countries in Europe and North America, very little is known about the health and healthcare use practices of uninsured residents, asylum seekers, and undocumented migrant workers in Israel. In this study, we describe the demographic characteristics and clinical visits of asylum seekers and undocumented migrant workers at Terem Refugee Clinic (TRC), a clinic in Tel Aviv available only to uninsured residents of Israel, from 2013 through 2017. 

## 2. Materials and Methods 

This serial cross-sectional study was conducted using visit data from TRC, a clinic located in the Tel Aviv Central Bus Station, which was opened in 2013 in order to serve exclusively asylum seekers and other undocumented, uninsured residents in Israel. Terem is a private organization that operates a network of urgent care centers in Israel, nearly all of which serve the general Israeli population, who are insured under Israel’s system of universal coverage. TRC is an exception, funded by the Government of Israel through a contract from the Ministry of Health to serve the uninsured. In order to receive care at TRC, patients must show identification specific to asylum seekers or migrant workers. For asylum seekers, this identification is a conditional release document from the Ministry of the Interior. For migrant workers, this identification is a passport with an expired working visa. Treatment is not provided to Israeli citizens or tourists. 

Terem provides the staffing and infrastructure for the urgent care clinic, including an electronic medical record system, evidence-based protocols for treatment of frequent diagnoses, point-of-care laboratory and radiology services, diagnostic equipment, and treatment supplies. The TRC, like many urgent care clinics, provides services including and beyond what may be available at most general practitioners’ offices, but short of what is typical for a hospital-based emergency department. Diagnostic services include vital signs, doctor’s examination, point-of-care laboratory, electrocardiography, radiology, and limited ultrasound. Treatment services include oral and parenteral medicines, respiratory inhalations, suturing, incision-drainage and similar bedside procedures, and casting. Unavailable at TRC are CT scans, magnetic resonance imaging, and thrombolytics, and patients who need services such as these are sent to one of two local hospitals. For every urgent care visit, patients pay a flat rate of 20 NIS (approximately USD 5.50), which includes triage with vital signs taken by a paramedic, a doctor’s examination, blood tests, radiology imaging, and bedside procedures as clinically indicated. While patient country of origin is not recorded as part of routine clinic visits, based on the staff’s experience at TRC, the vast majority of patients at TRC are Eritrean, with Sudanese a distant second.

We evaluated the electronic medical records for all patient visits at TRC from 1 January 2013 through 31 December 2017. For each medical visit, we extracted data on the patient’s age, sex, discharge status, and diagnosis.

At the end of every patient visit at TRC, doctors choose at least one diagnosis from a list of 623 possible diagnoses (see Supplementary Table S1). In order to better understand the main reasons for patient visits, we matched each diagnosis with its equivalent ICD-10 code and then assigned each of the 623 diagnoses to one of 22 diagnostic categories. These 22 categories were largely based on ICD-10 body-system categories that were used in a previous study of healthcare in refugees in Switzerland [7]. Some ICD-10 organ-system categories, such as respiratory diseases and digestive system diseases, include acute infections, such as acute respiratory infections and gastroenteritis (Supplementary Table S1). For discharge diagnoses that did not fit clearly into one of the 22 disease categories, two TRC medical doctors (O.Z., M.K.) agreed on the appropriate disease category by consensus. In order to not count individual patient visits multiple times if the visits were associated with multiple diagnoses, we only included the first diagnosis in our main analysis. However, we also performed a separate analysis that included all diagnoses to evaluate for significant differences in trends reported in this paper. 

Doctors at TRC refer patients presenting with symptoms suspicious for tuberculosis (TB) or patients with exposure to known TB cases to a government-funded, regional TB center in Israel, where more extensive TB testing and treatment is offered. In addition, because TRC is an urgent care clinic without all the services of a hospital emergency room, doctors refer patients who need more intensive medical treatment to an area hospital, sometimes by ambulance. As part of our analysis, we also examined whether patient visits ended with a referral to the regional TB clinic or a discharge to the hospital. 

We analyzed data for changes in the age and gender distribution of patients treated at the clinic over time, changes in the percentage of patients discharged to hospital and referred to tuberculosis clinic over time, and changes in top diagnosis groups over time. The unit of time used in the analysis was the calendar year. We used a chi-squared test for trends (Cochran–Armitage test for trend) to determine statistical significance. A *p*-value less than 0.05 in the chi-squared test for trends indicated that the trend in the variable from 2013 to 2017 was statistically significant. R version 3.4.3 was used for this analysis. 

The Ethical Review Committee of Ben-Gurion University of the Negev approved this study.

## 3. Results

### 3.1. Overall Patient Visits and Age Distribution

Over the five years, there were 99,569 visits to TRC. Visits were lowest in 2013 (11,112) and relatively stable from 2014–2017 (range: 19,712–23,172). Overall, most clinic visits were among adults aged 18–35 (41.2%) and among children under 2 years of age (23.7%). Relatively few clinic visits occurred in patients older than 50 years of age (3.0%) (Table 1). Over the five years, the age distribution of patient visits varied slightly. The percentage of patients under 2 years old and patients 18–35 years old decreased from 2013 to 2017, while the percentage of patients 2–5 years old, 5–18 years old, 35–50 years old, and 50 years old and older increased.

### 3.2. Gender

Among all clinic visits, 44,580 (44.8%) were among females and 53,989 (55.2%) were among males. The overall male:female ratio for all visits was 1.1 for children under 18 years old and 1.3 for adults. Although the male:female ratio for children did not change significantly over time, the male:female ratio for adults decreased from 1.7 in 2013 to 1.1 in 2017 (*p* for trend <0.00001).

### 3.3. Hospital and TB Clinic Referrals

Overall, 2359 (2.4%) of all patient visits ended in a hospital referral (Table 1). The percentage of patients referred to the hospital varied from a low of 1.9% in 2015 to a high of 3.3% in 2017 (*p* for trend <0.00001). Over the five years, the most common diagnoses for patient visits that ended in hospital referral were upper respiratory infection (11.7%), cough (4.5%), unspecified abdominal pain (3.6%), and bronchitis (3.0%). The median age of patients referred to the hospital was 25.5 years (interquartile range 2.2–32.5) and most patients (55.6%) were male. 

Over the five years, 498 (0.5%) of all patient visits ended with a referral to the regional TB clinic (Table 1). The percentage of patients referred to the TB clinic for further diagnostics ranged from a low of 0.2% in 2014, to a high of 0.9% in 2017 (*p* for trend <0.00001). The median age of patients referred to the TB clinic was 26.2 (IQR 2.4–33.0) and 282 (56.6%) were male.

### 3.4. Diagnositic Categories 

**Figure 1:**
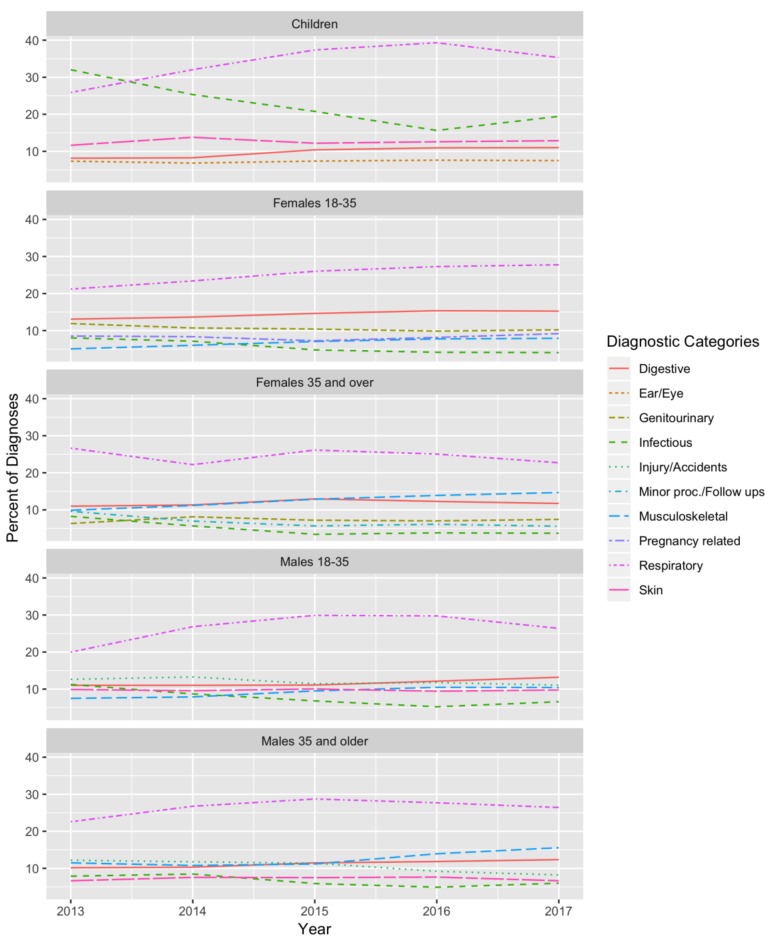
Percent of total diagnoses in each diagnostic category by age and gender over time.

For the five-year period, the most common diagnoses for children were respiratory diseases (35.1% of diagnoses), infectious diseases (21.4%), skin diseases (12.7%), digestive system diseases (10.0%), and eye and ear diseases (7.3%) (Table 2). These diagnostic groups did not differ by gender for children. Compared to younger children, children older than 5 years old had a higher percentage of injuries and accidents, which was the third most common diagnosis in children over 5 years old (21.5%). The percentage of diagnoses in children categorized as respiratory diseases increased from 25.9% in 2013 to 35.3% in 2017 and overtook infectious diseases as the top diagnosis group in 2014 (*p* for trend <0.00001). Conversely, the percentage of infectious disease diagnoses in children decreased over time, from 32.0% in 2013 (the most common diagnosis) to 19.4% in 2017 (the second most-common diagnosis) (*p* for trend <0.00001) (Figure 1).

In contrast to children, over the five-year period, adults 18 years of age and older were most commonly diagnosed with respiratory diseases (26.3%), digestive system diseases (12.7%), musculoskeletal diseases (9.6%), injuries and accidents (8.5%), and skin diseases (7.5%). There were differences in the most common diagnostic groups between men and women, but respiratory diseases were the top diagnostic group across all age groups of men and women (Table 2). Women aged 18 to 35 were commonly diagnosed with genitourinary diseases (10.5%) and pregnancy-related conditions (8.3%). Women aged 35 and older were also commonly diagnosed with genitourinary conditions (7.3%). Men were most commonly diagnosed with conditions relating to injury and accidents (12.0% in males 18–35 and 10.1% in males 35 and older); for women, these diagnoses were much less common.

As in children, in adults, the percentage of diagnoses categorized as infectious diseases decreased from 2013 to 2017 for both males and females in all age groups (*p* for trend <0.005 for all) (Figure 1). By contrast, the percentage of diagnoses categorized as digestive system diseases and musculoskeletal diseases increased from 2013 to 2017 for both males and females in all age groups (*p* for trend <0.01 for all), with the exception of digestive system diseases in women aged 35 and older, where it remained the third most common diagnosis over time.

Of the 99,569 patient visits, 13.6% had more than one diagnosis recorded. For reasons described in the above methods section, our main analysis included only the first of these diagnoses when counting the frequency of diagnostic groups. When we analyzed the data including all diagnoses per patient instead of including only the first one, the top five diagnosis groups in children and in adults remained the same, with two exceptions: In women 18–35 years old, pregnancy-related conditions, which were the fourth-most common diagnosis category in the main analysis, became the third most common diagnostic category, and genitourinary diseases, which were the third-most common diagnosis category, became the fourth most common diagnosis (data not shown).

## 4. Discussion

In our study, the first to describe the trends in demographics and medical visits of asylum seekers and uninsured migrant workers in Israel, at the largest urgent care clinic in the country serving this population, we found that over a five-year period, most people who came to TRC were children less than 2 years old and young adults. Over time, patients presented to the clinic with fewer infectious diseases overall, and more musculoskeletal diagnoses. In addition, there were more respiratory and, to a lesser extent, digestive system diagnoses. By 2017, the disease visit profile at TRC was quite similar to disease visit profiles described in other clinics in Israel that serve the general Israeli population [13].

The trends from diagnostic categories data showed evidence of a decreasing burden of infectious diseases among asylum seekers and undocumented migrants. We found that over time, the percentage of overall infectious disease diagnoses decreased in both children and adults. However, there was a relative increase in respiratory and digestive diseases, which include some acute infections, but this increase was considerably less than the decrease in infectious diseases. During the same time period, there was an increase in the relative percentage of diagnoses more reflective of chronic, noncommunicable illnesses, such as musculoskeletal disease in adults. Medical treatment of refugees and immigrants frequently focuses on treating infectious diseases, and screening and care for chronic disease conditions is often limited [12]. This focus leads to an increased burden of undiagnosed and untreated chronic diseases, as these conditions worsen and require more intensive treatment [14]. In addition to the chronic diseases that refugees and immigrants already have on arrival to their new country, shifts in lifestyle, such as changes in diet and lack of exercise, can lead to the development of new chronic diseases in this population [15]. Currently, there is minimal governmental support in Israel for longitudinal ambulatory care of undocumented residents with chronic diseases. However, our findings suggest that chronic diseases are a large, growing problem in this population. If more resources were dedicated to this problem, this community would likely be healthier and have fewer long-term complications, and costs to the overall healthcare system would be alleviated in future years. 

The clinic had a relatively low overall percentage of hospital referrals (2.3%) over the five-year period. This percentage of hospitals referrals at the TRC clinic was much lower than the percentage of hospital referrals cited in a previous study that described data from 2008 to 2011 from public clinics serving uninsured asylum seekers and uninsured migrant workers in Israel (40%) [16]. However, that study included data from clinics which have limited onsite diagnostic and treatment capacity compared to TRC. The fact that the clinic is equipped to perform on-site lab testing, X-rays, casting, intravenous treatments, and other interventions likely reduces hospital referrals and in turn reduces potential hospital costs among a population with very limited ability to pay large hospital bills. Therefore, the relatively low number of hospital referrals in our study is likely a reflection of the greater treatment capacity at TRC and is unlikely to indicate improved health in the population over time. The increase in hospital referrals found in 2017 compared to all prior years may reflect a change in the internal TRC guidelines for hospital referrals, which were implemented in 2016.

Throughout the five-year period, a high percentage of clinic visits among males 18 years and older was related to injuries and accidents. This finding was also described in the refugee population in Switzerland [7]. Asylum seekers and undocumented migrants have limited legal status, and therefore limited employment options. Migrant workers and asylum seekers often take jobs with working conditions that are hazardous and unsafe, leading to injuries and accidents [7,16]. In Israel, many asylum seekers and undocumented migrant workers are employed in restaurants, construction sites, and cleaning—settings which can be prone to injuries.

Tuberculosis continues to cause high levels of morbidity and mortality globally. The vast majority of patients at TRC are from two countries, Eritrea and Sudan, where TB is endemic [17,18]. In Israel, all asylum seekers who arrived through the Sinai Peninsula were screened for TB upon arrival with a Mantoux test, and referred to free, government-funded TB treatment centers [19]. Factors like language barriers, lack of familiarity with the medical system, and suboptimal case reporting and tracking infrastructure are known barriers to TB care and treatment in other settings [20] and may be relevant barriers to TB care in the asylum seeker and migrant worker populations in Israel. In our study, a small percentage of patient visits were referred from TRC to the regional TB treatment center every year. TRC does not receive regular communication about whether referred patients are actually seen at the TB center, and, if so, whether TB was confirmed and treated. Thus, potential TB infections in the population presenting to TRC are likely the result of patients not receiving proper treatment upon entry due to barriers to care, reactivation of latent TB, or TB infection acquired in Israel. 

Every year from 2014 to 2017, there were progressively fewer children under 2 years of age presenting to TRC, as a percentage of all clinic visits per year. This change could be a result of more parents purchasing health insurance for their very young children. A change in Israeli health care policy in 2016 lowered premiums for children’s health insurance regardless of their parents’ legal status in Israel [21]. This decrease in cost could have led more asylum seekers and uninsured migrant workers to enroll their children in a health insurance plan through one of the existing national health funds operating within the Israeli National Health Insurance Law.

Over the five-year period, fewer young adults aged 18 to 35 attended the clinic. This demographic change could reflect the ongoing process of “voluntary” migration from Israel encouraged by the Israeli government. According to informal interviews with NGOs working with asylum seekers in Israel, the healthiest individuals in the population preferentially take on migration. 

Finally, over the five-year period, relatively higher percentage of women used the clinic compared to men, which may reflect the fact that more men are receiving health insurance through work. In addition, more women may be using the clinic for reproductive health needs, including pregnancy care and contraception consultations. Finally, this trend may reflect an increase in the number of men leaving Israel through resettlement campaigns. 

## 5. Conclusions

This study was able to use a robust electronic medical record (EMR) system to evaluate trends in the healthcare use of asylum seekers and undocumented migrant workers in Israel. Because the EMR has been in place since the clinic opened in 2013, we were able to evaluate trends from the clinic’s first year for a five-year period. 

This study had limitations, which were mostly related to the availability of data and the coding of the data. We grouped discharge diagnoses by ICD-10 disease categories, some of which include some overlap between infectious and chronic diseases; for example, some infectious diseases, such as acute upper respiratory infection, are included in an ICD-10 organ system disease category, respiratory diseases. This approach to coding makes it more difficult to draw conclusions about trends in infectious relative to chronic diseases. While there was no change in coding instructions for physicians at the clinic that would explain varying disease trends, the broad nature of the ICD-10 categories limits our ability to draw broad conclusions about disease trends. 

TRC does not collect information on country of origin and time spent in Israel and, therefore, we were not able to evaluate the relationship of these two variables to trends in clinic visits. We were not able to link patient visits to individuals and, therefore, we could not determine how many times the same individual went to the clinic. In addition, our study population may not accurately represent the greater population of asylum seekers and undocumented migrants in Israel, due to its location in Tel Aviv and the convenience sample of patients treated at TRC. In addition, our study population may not accurately represent the entire population of asylum seekers and undocumented migrants in Israel. Some asylum seekers and undocumented workers in Tel Aviv and in Israel likely receive their medical care in clinics other than TRC. This study serves as one snapshot of the medically attended healthcare trends of this population. We did not have access to data from urgent care clinics attended by Israeli citizens and legal residents in Tel Aviv or elsewhere in Israel. Therefore, we could not compare trends in demographics or discharge diagnoses between the population that attended the Terem Clinic and the general Israeli population—citizens and legal residents—attending other clinics in in Tel Aviv or Israel. Undertaking this kind of comparison would be an important future direction for research. 

Additionally, because we lacked follow-up data on TB referrals, we could not evaluate trends in the incidence of TB among patients at TRC. The ICD-10 disease categories we used do not clearly distinguish between infectious and non-infectious diseases. For example, some acute respiratory and gastrointestinal infections are included in the ICD-10 disease categories of respiratory system diseases and digestive system diseases, respectively, and are therefore not included in our definition of infectious diseases. Trends in demographics used a chi-squared test for linear trend analysis that may have reported significance due to large sample size instead of due to robust evidence of a linear trend. Some of the trends reported may be better fit by a nonlinear trend.

Asylum seekers and undocumented migrant workers presented to TRC with decreasing infectious disease diagnoses and increasing chronic diseases diagnoses from 2013 to 2017. Future research on asylum seeker and undocumented migrant health should include data from other clinics and hospitals serving this population in Israel.

## Figures and Tables

**Table 1:** Patient characteristics and medical visit outcomes at Terem Refugee Clinic, 2013–2017.

Population Attributes	Total N (%)	2013 N (%)	2014 N (%)	2015 N (%)	2016 N (%)	2017 N (%)	*p* ^Ŧ^
Total	99,569	11,112	19,712	23,172	22,673	22,900	
Age ^1^							
Under 2 years	23,593 (23.7)	2928 (26.3)	5803 (29.4)	6197 (26.7)	4721 (20.8)	3944 (17.2)	<0.001
2–5 years	13,224 (13.3)	828 (7.4)	1992 (10.1)	3217 (13.9)	3423 (15.1)	3764 (16.4)	<0.001
5–18 years	4450 (4.5)	344 (3.1)	697 (3.5)	1000 (4.3)	1065 (4.7)	1344 (5.9)	<0.001
18–35 years	41,038 (41.2)	5366 (48.3)	8414 (42.7)	9396 (40.5)	9225 (40.7)	8637 (37.7)	<0.001
35–50 years	14,299 (14.4)	1388 (12.5)	2368 (12.0)	2847 (12.3)	3504 (15.4)	4192 (18.3)	<0.001
50 and over	2955 (3.0)	256 (2.3)	434 (2.2)	513 (2.2)	733 (3.2)	1017 (4.4)	<0.001
Male:Female ^2^ Children Adults	1.1 1.3	1.1 1.7	1.1 1.6	1.1 1.4	1.1 1.2	1.1 1.1	n.s. <0.001
Referred to hospital	2359 (2.3)	260 (2.3)	411 (2.1)	441 (1.9)	500 (2.2)	747 (3.3)	<0.001
Referred to TB clinic	498 (0.5)	58 (0.5)	47 (0.2)	89 (0.4)	101 (0.4)	203 (0.9)	<0.001

^Ŧ^*p*-value calculated using chi-squared test for trend in proportions. Trend was defined as changes in percentages in each category over the time, 2013–2017. Denominator used is the total number of patient visits that year denoted at the top of the column. Not calculated for age. ^1^ Age groups are inclusive at the low end and exclusive at the high end. ^2^ Denominator used for trends for ratio male:female was the total number of patient visits that year for children and the total number of patient visits that year for adult. TB: Tuberculosis. “n.s.” means non-significant, indicating a *p*-value > 0.05.

**Table 2:** Top five diagnostic categories of Terem Refugee Clinic patient visits, 2013–2017.

Rank	Children * <18 Years Old N (%)	Adults 18–34		Adults 35 and Older	
		Females N (%)	Males N (%)	Females N (%)	Males N (%)
Total	41,267	19,782	21,256	5242	12,012
1.	Respiratory 14,490 (35.1)	Respiratory 5085 (25.7)	Respiratory 5750 (27.1)	Respiratory 1268 (24.2)	Respiratory 3219 (26.8)
2.	Infectious 8827 (21.4)	Digestive 2888 (14.6)	Injury/Accidents 2555 (12.0)	Musculoskeletal 699 (13.3)	Musculoskeletal 1566 (13.0)
3.	Skin 5241 (12.7)	Genitourinary 2068 (10.5)	Digestive 2484 (11.7)	Digestive 692 (12.0)	Digestive 1377 (11.5)
4.	Digestive 4123 (10.0)	Pregnancy 1636 (8.3)	Skin 2070 (9.7)	Genitourinary 382 (7.3)	Injury/Accidents 1217 (10.1)
5.	Eye and Ear 3027 (7.3)	Musculoskeleta l1,387 (7.0)	Musculoskeletal 1961 (9.2)	Minor Procedures and Follow-Ups 325 (6.2)	Skin 866 (7.2)

* Data for children under 18 years old are combined for males and females.

## Supplementary Materials

The following are available online at http://www.mdpi.com/1660-4601/16/2/252/s1, Table S1: Terem diagnoses categorized based on ICD-10 system.
