# Supplementary material for: For What Illnesses Do Asylum Seekers and Undocumented Migrant Workers in Israel Seek Healthcare? An Analysis of Medical Visits at a Large Urgent Care Clinic for the Uninsured in Tel Aviv
Source: Int J Environ Res Public Health. 2019 Jan 16;16(2):252. doi: 10.3390/ijerph16020252 (PMC6352240; doi:10.3390/ijerph16020252)
Supplement: Supplementary file 1 [file ijerph-16-00252-s001.pdf]

Supplementary Table 1: Terem diagnoses categorized based on ICD-10 system

Diagnostic Groups*	Terem Diagnosis
A and B<br>Infectious and parasitic disease	Cat-scratch disease, Diarrhea Infectious, Dysentery Infectious colitis enteritis, Erysipelas, Gangrene, Gastroenteritis, Meningitis/Encephalitis Viral, Pertussis, Scarlet Fever, Septicemia, STD, TB related illness, Viral infection, Balanitis, Infection any, Chicken pox, Fungal GU infection,<br>Fungal infection any, Hand foot and mouth disease, Herpes genital, Herpes Simplex, Herpes Zoster, Lice head, Measles, Molluscum contagiosum, Mononucleosis, Mumps, Parasite/worms/pinworms, Rash viral, Scabies, tinea capitis, tinea corporis, tinea cruris, tinea pedis, Varicella uncomplicated, Wart, Chills, Fever nonspecific, IV ATBS (intravenous antibiotics)
C and D00-D48<br>Neoplasms	Malignant CA, Swelling/Mass in Abdomen, Swelling/Mass in head/neck, Lipoma
D50-D89<br>Diseases of blood and blood forming organs and disorders of immune mechanisms	Anemia, Blood disease, Henoch Schonlein purpura/allergic purpura, Leukocytosis, Neutropenia/Leukocytopenia, Pancytopenia, Purpura, Thrombocytopenia, Epistaxis nosebleed, Lymph nodes enlargement, Lymphadenopathy, Petechiae, Arteritis (temporal or any vasculitis), Coagulation management
E<br>Endocrine, nutritional, and metabolic diseases	Dehydration, DKA diabetic ketoacidosis, Goiter, Hyperthyroidism, Hypocalcemia, Hypoglycemia, Hyponatremia, Hypothyroidism, IDDM, IDDM with an acute complication, NIDDM Diabetes, NIDDM with acute complication Diabetes, Polycystic ovaries, Thyroid disorder nonspecific, Failure to thrive-child, Hyperglycemia, Intravenous fluids/treatment any, IVF treatment (intravenous fluid treatment)
F<br>Mental and behavioral disorders	Alcohol intoxication, Anxiety, Bipolar disorder/Manic-Depression, Dementia, Depression, Drug withdrawal, Globus Hystericus, Irritable/Agitation, Psychosis, Schizophrenia, Suicidal ideation, Insomnia
G<br>Nervous system diseases	Bell's palsy, Carpal tunnel syndrome, Migraine, Neurological disorder central, Peripheral neuropathy, Seizure, Sleep disturbance, Tension headache, TIA, Nystagmus, Spinal cord abnormality, Confusion Altered mental state, Dysarthria / speech disturbance, Febrile convulsions, Headache, Numbness, Syncope, Vertigo, Paresthesia

H<br>Eye and ear diseases	Blepharitis, Cerumen impacted, Chalazion, Discharge from ear, Otorrhea, Ear disorder nonspecific, Ear pain nonspecific, External ear disorder, Eye disorder, Glaucoma, Hearing loss, Mastoiditis, Otitis externa, Otitis media, Otitis Media serous, Pain eye/periocular nonspecific, Perforated tympanic membrane, Periorbital/Orbital cellulitis, Retinal abnormality, Stye/Hordeolum, Subconjunctival hemorrhage, Tinnitus, Visual disturbance acute, Welders Keratitis, Conjunctivitis, Discharge from Eye
I<br>Cardiovascular diseases	ACS (acute coronary syndrome), Acute MI, Angina Pectoris, Arrhythmia, Atrial fibrillation, Atrial flutter, CVA, DVT, Endocarditis, Heart disease, Hypertension, Hypotension, Lymphadenitis acute, Pericarditis, Peripheral vascular disease, Premature beats, Raynaud's syndrome, Varicocele, Varicose veins/acute bleeding, Venous (peripheral) insufficiency, Venous (stasis) ulcer, CHF congestive heart failure, Cardiac murmurs, Palpitations, Tachycardia, Bradycardia, Chest pain, EKG nonspecific changes, Superficial thrombophlebitis
J<br>Respiratory system diseases	Apnea, Asthma exacerbation, Bronchiolitis, Bronchitis, COPD - Chronic bronchitis, Epiglottitis acute, Flu, Inhalation of fumes/gas/economica, Laryngitis/Croup, Pharyngitis, Pleural effusion, Pneumonia, Pneumothorax, Respiratory failure, Rhinitis Acute Nasopharyngitis, Seasonal allergy, Sinusitis, Tonsillitis, URI nonspecific, Cough, Dyspnea, Hemoptysis, Postnasal drip, Stridor, Tachypnea, Voice change / hoarse, Wheezing, Abscess deep pharyngeal, Croup (in children), Discharge from nose, Hypoxia/Hypoxemia, Mediastinal disease
K<br>Digestive system diseases	Tongue lesion/abnormality, Anal fissure, Aphthous ulcer, Appendicitis, Aphthous ulcers in mouth, Bleeding GI, Blood in stool/Hematochezia, Bowel obstruction, Cholecystitis, Cholelithiasis, Constipation, Dental abscess, Dental caries, Dental disorder any, Dental trauma, Dental/gingival infection, Esophageal disorder, Esophageal reflux GERD, Esophagitis, Gastritis, Gingival abscess/infection, Glossitis, Hematemesis, Hemorrhoids, Hernia, Inflammatory bowel disease IBD, Intestinal obstruction, Intussusception, Irritable Bowel Syndrome (IBS), Melena, Oral disease nonspecific, Peritonitis acute abdomen, PUD (peptic ulcer disease), Salivary duct stone (Sialolithiasis), Salivary gland infection, Sialadenitis (includes Parotitis), Stomatitis, Teething, TMJ disorders, Ulcerative colitis/Crohn's disease, Abdominal pain nonspecific, Blood in stool non-specific, Dyspepsia, Dysphagia, Flatulence Burping Eructation, Hiccup/singultus, Incontinence of feces,

	Jaundice, Nausea nonspecific, Pain in throat nonspecific, Pain perirectal/perianal/anal, Vomiting, Diarrhea, Diverticulitis, Gastric band deflation/inflation, Hepatitis/Jaundice, Pancreatic disease, Rectal anal disease any
L<br>Skin diseases	Abscess Furuncle, Acne, Atopic dermatitis, Cellulitis decubitus ulcer, Dermatitis, Dermatitis eczema, Erythema multiforme, Felon, Folliculitis, Hair loss alopecia, Impetigo, Ingrown Toenail, Nail disorder/disease any, Paronychia/nail disorders, Pilonidal cyst, Pruritus, Psoriasis, Rash Diaper/Friction/Heat, Rash drug induced, Sebaceous cyst, Skin irritation/dermatitis from chemical, Skin irritation/dermatitis from drug, Skin irritation/dermatitis from soap, Skin lesion benign, Skin ulcer chronic, Sunburn, Urticaria, Ecchymosis Bruise, Any Rash, Itching
M<br>Musculoskeletal diseases	Achilles tendonitis, Arthritis - Monoarthritis, Arthritis - Polyarthritis, Arthritis bacterial/septic, Arthritis Infectious, Arthritis Rheumatoid, Arthritis viral, Articular disease/Arthritis, Back pain, Baker's cyst, Bone cyst, Bursitis, Bursitis prepatellar, Calcaneal Spur, Calcific bursitis/tendinitis Shoulder, Chondritis/Perichondritis of ear, Coccygodynia, Costochondritis, Discopathy Lumbar, Dislocation of shoulder recurrent, Enthesopathy Knee, Exostosis, Fasciitis any, Flat Foot, Ganglion of joint, Gout, Hemarthrosis, Joint contracture, Joint effusion/hemarthrosis, Joint pain (also post traumatic), Ligament laxity, Limp, Mallet finger, Muscle Ligament Disorder, Muscle spasm, Musculoskeletal Symptoms, Myalgia, Neck pain/Cervicalgia, Neuralgia, Olecranon bursitis, Osteoarthritis, Osteomyelitis, Pain in joint nonspecific, Pain in limb nonspecific, Plantar fasciitis, Rhabdomyolysis, Rotator Cuff disease, Sciatica, Scoliosis, Synovitis, Tendinitis, Tendon rupture, Tendon Rupture - Extensor Digital, Tendon Rupture - Flexor Digital, Tennis Elbow, Tenosynovitis of the foot/ankle, Tenosynovitis of the hand/wrist, Torticollis or any MSK neck disease
N<br>Genitourinary diseases	Amenorrhea, Bartholin Abscess/Cyst, BPH, Breast lump or mass, Cystocele, Dyspareunia, Epididymitis/Orchitis, Genital problem nonspecific, Gynecomastia, Hematuria, Hydrocele, Mastitis, Mastodynia Breast Pain, Menorrhagia, Menstrual disorder, Metrorrhagia, Nephritis, Ovarian cyst, Penile disorder, PID/pelvic inflammatory disease, Prolapse of uterus, Prolapse of vaginal walls, Prostatitis, Proteinuria, Pyelonephritis, Renal colic, Renal failure, Renal failure acute, Renal failure chronic, Testicular pain, Testicular torsion, Urethritis, Uterine Myoma, UTI, Vaginal bleeding, Vaginal bleeding Dysmenorrhea, Vaginal Discharge, Vaginal pain, Vaginitis,

	Vulvar infection/inflammation, Discharge from Urethra, Dysuria, Nocturia, Polyuria, Urinary complaints, Urinary frequency, Urinary incontinence, Urinary retention, Urinary stream slow, Urinary urgency, Urinary catheter or stoma check and/or adjustment, Urinary catheter placement, Urinary catheter removal, Urinary catheter replacement, Asymptomatic bacteriuria, Bleeding (vaginal), Gynecologic Exam/procedure, Removal device (eg IUD) from GU tract
O<br>Pregnancy related conditions	Abruptio Placenta, Asymptomatic bacteriuria in pregnancy, Braxton Hicks contractions, Decreased fetal movements, Delivery, Ectopic pregnancy, Hyperemesis gravidarum, Late vomiting of pregnancy, Missed abortion, Obstetric trauma, Post term pregnancy, Pre/Eclampsia, Pregnancy, Pregnancy - complication, Pregnancy uncomplicated, Premature rupture of membranes, Spontaneous abortion complicated, Spontaneous abortion uncomplicated, Threatened abortion, Umbilical cord hemorrhage, Uterine contractions, Contraceptive management, Contraceptive management emergency, Breast inflammation/infection, S/P cesarean delivery
P<br>Conditions originating in perinatal period	Feeding problem, Omphalitis, Postpartum care - lactation, Crying excessive (in infant)
Q<br>Congenital malformations, deformations and chromosomal abnormalities	Congenital hip dislocation
R<br>Symptoms, signs and abnormal clinical and laboratory findings, not elsewhere classified	Dizziness, Pain facial, Weakness / Malaise / Fatigue / Apathy, Edema, Pain
S and T<br>Injury and accident	Any abrasion, Abrasion arm, Abrasion hand, Abrasion head, Abrasion hip leg, Abrasion trunk, Amputation finger, Amputation thumb, Animal bite, Blister, Concussion with LOC/ Head Trauma, Concussion without LOC/ Head Trauma, Contusion, Contusion of abdomen, Contusion of ankle, Contusion of arm, Contusion of back, Contusion of buttock, Contusion of chest, Contusion of elbow, Contusion of face, Contusion of finger, Contusion of foot, Contusion of forearm, Contusion of hand, Contusion of head, Contusion of hip, Contusion of knee, Contusion of leg, Contusion of shoulder,

	Contusion of toe, Contusion of upper limb, Contusion of wrist, Crush injury, Dislocation, Dislocation of acromioclavicular joint, Dislocation of elbow, Dislocation of finger, Fx, Fx calcaneus, Fx carpal bone, Fx clavicle, Fx coronoid process, Fx cuboid, Fx cuneiform, Fx distal phalanx, Fx distal phalanx - Open, Fx facial bone, Fx femur, Fx femur - Low End, Fx femur - Neck, Fx fibula, Fx foot bone, Fx humerus, Fx humerus - Greater Tuberosity, Fx humerus - Lateral Condyle, Fx humerus - Medial Condyle, Fx humerus distal (supracondylar), Fx Lower Forearm, Fx Lumbar Vertebra, Fx Lunate, Fx Malleolus - Lateral, Fx Mandible, Fx medial malleolus, Fx metacarpal, Fx Metatarsal, Fx nasal bone, Fx Navicular - Foot, Fx Olecranon, Fx open, Fx patella, Fx pelvis, Fx phalanx hand, Fx Pisiform, Fx Radial Neck, Fx Radius - Shaft, Fx radius distal, Fx radius head, Fx rib, Fx scaphoid, Fx skull closed, Fx talus, Fx tibia, Fx Toe, Fx Trapezium, Fx Triquetrum, Fx Ulna - Upper End, Fx Ulna -Shaft, Fx ulna distal, Fx vertebral, head Injury, Injury finger, Injury Hand, Injury lung, Laceration back, Laceration face, Laceration finger, Laceration foot, Laceration forearm, Laceration hand, Laceration leg, Laceration neck, Laceration thigh, Laceration upper arm, Sprain, Sprain interphalangeal joint, Sprain knee, Sprain of ankle, Sprain of foot, Sprain of hand, Sprain of knee or leg, Sprain of neck, Sprain of wrist, Sprain shoulder, Sprain sternoclavicular joint, Tooth broken, Animal attack, Abuse adult, Abuse child, Allergic reaction, Anaphylaxis, Angioedema, Burn 1st degree any, Burn 2nd degree any, Burn 2nd degree face, Burn of face/head, Burn third degree, Burn to arm, Burn to leg, Burn to trunk, Corneal disorder abrasion foreign body, Food poisoning, Foreign body anus rectum, Foreign body in airway, Foreign body in arm, Foreign body in ear, Foreign body in esophagus, Foreign body in finger, Foreign body in foot, Foreign body in GI tract, Foreign body in hand, Foreign body in head, Foreign body in hip or leg, Foreign body in intestine (colon), Foreign body in leg, Foreign body in nose, Foreign body in pharynx, Foreign body in trunk, Foreign body of eye, Foreign body in vagina, Human bite, Ingestion of substance toxic/non-toxic, Insect bite/sting, Jellyfish/marine animal sting, Overdose Acamol or ASA, Overdose drug, Poisoning, Post-op/procedure complication, Wound open infected, Electrical injury, Assault, Hematoma, Laceration - adhesive closure, Laceration - bandage, Laceration - suture, Pulled elbow, Removal of foreign body, Side effect of medication
V and Y<br>External causes of morbidity and mortality	Traffic Accident, MVA (motor vehicle accident), Needle stick accidental, Fall, Assault physical

Z<br>Minor procedures and follow ups	Tetanus vaccination, Bandage change for a wound, Cast check, Prescription, Suture removal, Ultrasound exam, Examination any, Follow-up exam, General medical exam, Injection/infusion of medication, IV access, Laboratory Exam, Normal examination, Vaccination for flu, Vaccination/Immunization, Wound care, X-Ray only

*Diagnostic groups were formed using the ICD-10 system. Each Terem diagnosis was categorized into ICD-10 blocks, and then similar ICD-10 blocks were grouped together to form the diagnostic categories used in analysis. For example, ICD-10 blocks A and B are both infectious disease, so they were combined into one diagnostic category. Similarly, ICD-10 blocks S and T are both injuries and accidents so they were also combined into one diagnostic category.
